# MAS: Standalone Microwave Resonator to Assess Muscle Quality

**DOI:** 10.3390/s21165485

**Published:** 2021-08-14

**Authors:** Viktor Mattsson, Leanne L. G. C. Ackermans, Bappaditya Mandal, Mauricio D. Perez, Maud A. M. Vesseur, Paul Meaney, Jan A. Ten Bosch, Taco J. Blokhuis, Robin Augustine

**Affiliations:** 1 Ångström Laboratory, Division of Solid State Electronics, Department of Electrical Engineering, Uppsala University, SE-75121 Uppsala, Sweden; viktor.mattsson@angstrom.uu.se (V.M.); bappaditya.mandal@angstrom.uu.se (B.M.); mauricio.perez@angstrom.uu.se (M.D.P.); 2 Department of Traumatology, Maastricht University Medical Centre+, 6229 HX Maastricht, The Netherlands; mam.vesseur@student.maastrichtuniversity.nl (M.A.M.V.); jan.ten.bosch@mumc.nl (J.A.T.B.); taco.blokhuis@mumc.nl (T.J.B.); 3 Thayer School of Engineering, Dartmouth College, Hanover, NH 03755, USA; Paul.M.Meaney@dartmouth.edu

**Keywords:** muscle quality, sarcopenia, microwave sensing, bandstop filter, substrate integrated waveguides

## Abstract

Microwave-based sensing for tissue analysis is recently gaining interest due to advantages such as non-ionizing radiation and non-invasiveness. We have developed a set of transmission sensors for microwave-based real-time sensing to quantify muscle mass and quality. In connection, we verified the sensors by 3D simulations, tested them in a laboratory on a homogeneous three-layer tissue model, and collected pilot clinical data in 20 patients and 25 healthy volunteers. This report focuses on initial sensor designs for the Muscle Analyzer System (MAS), their simulation, laboratory trials and clinical trials followed by developing three new sensors and their performance comparison. In the clinical studies, correlation studies were done to compare MAS performance with other clinical standards, specifically the skeletal muscle index, for muscle mass quantification. The results showed limited signal penetration depth for the Split Ring Resonator (SRR) sensor. New sensors were designed incorporating Substrate Integrated Waveguides (SIW) and a bandstop filter to overcome this problem. The sensors were validated through 3D simulations in which they showed increased penetration depth through tissue when compared to the SRR. The second-generation sensors offer higher penetration depth which will improve clinical data collection and validation. The bandstop filter is fabricated and studied in a group of volunteers, showing more reliable data that warrants further continuation of this development.

## 1. Introduction

Sarcopenia is a progressive skeletal muscle disorder with loss of muscle mass, strength, and function [1]. Sarcopenia represents an initial decrease in muscle mass and size, but eventually, there is a reduction in muscle tissue quality characterized by replacement of muscle fibers with fat, increased fibrosis, changes in muscle metabolism, oxidative stress and decreased muscle function [2,3]. Sarcopenia synergistically worsens the adverse effects of obesity in older adults, which results in sarcopenic obesity. A model has been proposed and developed that links the age-related interplay between adipose and muscle tissues, which is believed to contribute to the development of sarcopenic obesity [3]. Age-related sarcopenia leads to poor quality of life, risk of falls and fractures and increased mortality [3,4,5]. Recently, sarcopenia was shown to be a risk factor in vertebral compression fractures with 2.3 times increased risk for people with sarcopenia [6]. Sarcopenia also impairs the ability to perform daily activities and studies have shown that this may lead to higher health care costs [4,7]. Sarcopenia can also be a prognosis tool in the survival rate of cancer as skeletal muscle depletion has been shown to be a tool to prognose the survival rate of cancer [8]. In a recent study, women diagnosed with non-metastatic breast cancer and sarcopenia show a significantly increased risk of death than those diagnosed with the same type of cancer but not sarcopenia [9]. Since sarcopenia and muscle depletion might be associated with an increased risk of death, early identification of this disease is needed.

The European Working Group on Sarcopenia in Older People (EWGSOP) has come to a consensus to help diagnose and define sarcopenia [1]. Such consensus indicated that there are still many gaps in the knowledge about sarcopenia, the diagnostic tools and their cutoff points [5]. A wide variety of tools for diagnosing sarcopenia is available and each tool has specific advantages [1]. However, varying investigational settings make it difficult to adopt these tools systematically.

Currently, different techniques are available for quantification of muscle mass and function: hand-grip strength test (HGS), Bio Impedance Analysis (BIA), ultrasound (US), muscle area for computerized tomography (CT) scan at the third lumbar level (L3) and magnetic resonance imaging (MRI). In addition to these techniques, the EWGSOP mentions alternative methods such as gait speed, creatine dilution test, and questionnaires (Sarcopenia-Quality of Life (SarQoL) and SARC-F) [1]. Techniques such as hand-grip strength (HGS) [10] and gait speed can be used to test the strength and performance of the muscle which has an indirect relation to muscle mass.

The gold standard in the diagnosis of sarcopenia is the Skeletal Muscle Index (SMI) derived from the muscular area in a CT scan at the third lumbar level [1]. SMI has been shown to be significantly correlated to whole-body muscle quantity [11,12] and is an accurate predictor of sarcopenia [13,14]. Although techniques such as CT and MRI are accurate, they require expensive equipment. Significant disadvantages include inconsistent cut-off points in different studies, high medical costs, and the ionizing radiation burden. MRI does not use radiation and provides additional information on muscle quality. However, costs and contra-indications for MRI render it less suitable for the assessment of muscle mass in clinical practice. Handheld and ambulatory techniques such as BIA and ultrasound are easier to use but limited in accuracy or interobserver variability. Handgrip strength (HGS) is a good screening tool since it is easy to use and replicable, but lacks accuracy and does not give information about the muscle quality [1]. These shortcomings inform the need for tools that are widely available, user-friendly, efficient, non-invasive and inexpensive. Low-power microwave sensors and imaging systems for biomedical applications have an important advantage, over ionizing radiation techniques such as X-rays since microwaves are non-ionizing and less harmful. Therefore, microwave sensors have been used for breast cancer detection utilizing sensor arrays to create imaging systems [15,16]. For glucose measurements, a portable system has been developed using four complementary split ring resonators [17]. Glucose sensing is an area in which a lot of different sensors have been developed [18,19,20,21]. Microwave sensors developed on flexible substrate materials are also researched with the added benefit of having a sensor that is conformal to the body and not a rigid structure [22,23,24]. Also, the feasibility of using the fat channel for intra-body communication at microwave frequencies has been studied [25]. In vivo characterization using microwave sensors have been done in hip fracture healing [26], to monitor intracranial pressure [27], measure fluid volume changes in the heart [28] and measure limb hemodynamics [29]. Recently, non-invasive techniques to characterize the dielectric properties and analyze variations in biological tissues are being studied for their potential to determine muscle mass and quality [30]. In 2018, Mohd Shah et al. [31] published a depth assessment study exploring the level to which the electric field penetrates through skin, fat and muscle. A pre-clinical study was performed in laboratory with phantoms (synthetic materials emulating human tissues) and ex vivo porcine tissues studying the effect of tissue thickness variation [32]. Because these results were promising, we have proposed and are currently developing a tool to assess muscle quality via microwave characterization.

A system containing a microwave sensor and a small vector network analyzer (VNA) called The Muscle Analyzer System (MAS) was fabricated. It was designed to be a portable, easy-to-use device that could be used in a clinical setting for screening, diagnosis and follow up of sarcopenia.

**Figure 1:**
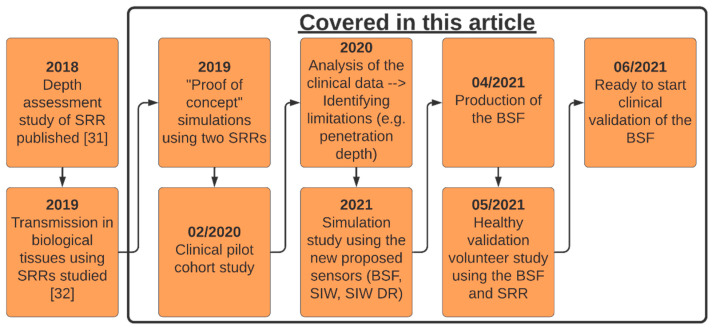
Illustrative timeline of previous works and the progress for the MAS device, BSF = Bandstop filter, SRR = Split-ring resonator, SIW = Substrate Integrated Waveguides sensor, SIW DR = SIW with dielectric resonator sensor.

This article presents a new technique for quantification of muscle mass. The process of designing and fabricating this device and its drawbacks is an important learning path. In this article, we aim to evaluate the potential of this technique and compare our approach to the SMI in a pilot cohort study. Alternative microwave sensors are evaluated via numerical simulations with the purpose of identifying a sensor that penetrates more signal to the muscle layer. Figure 1 is a definitive timeline of the work done previously with the split-ring resonator (SRR) and progress made with the MAS device to this point. The black box highlights the different parts of the work presented in this article.

## 2. Methods

### 2.1. The Sensing Principle

**Figure 2:**
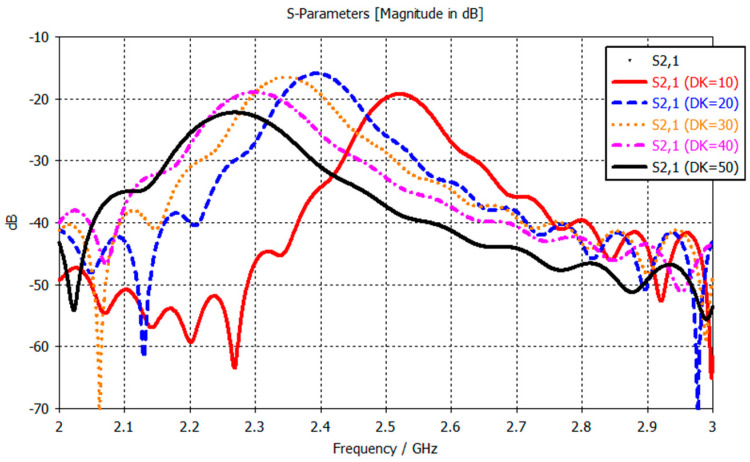
Frequency response for varying dielectric properties for the SRRs.

There are several different microwave sensing strategies, as outlined in [33], such as sensors based on coupling modulation [34], frequency splitting [35] and amplitude modulation [36,37]. The strategy we employ is using sensors based on variations in the frequency response because they can be used for sensing material characteristics, e.g., permittivity, and are typically low cost and simple designs [33]. A decrease in muscle quality, as seen in sarcopenia, results in increased intramuscular adipose tissue [38]. The high contrast between adipose tissue and muscle tissue properties at microwave frequencies could be exploited to detect changes in muscle quality, since there is more adipose tissue in between the muscle [13]. The primary rationale is that the resonance properties of the frequency response from the transmission coefficient, S_21,_ change depending on the composition of the material under the sensor. By changing the material beneath the sensor, we alter that area’s dielectric properties, which subsequently induces a change in the frequency response. Figure 2 shows the frequency response, and how it changes when the dielectric constant (DK) of the material under test changes (from simulations using two split-ring resonators placed 2 mm apart). The observed change is a resonance frequency decrease as the DK of the material increases, 2.52 GHz for DK 10 and 2.27 GHz for DK 50. This is a simple one-layer model, whereas in the human body, the signal from the sensors need to penetrate through the skin and fat layers into the muscle layer, where the changes in dielectric properties will occur. Therefore, the changes in the frequency response are expected to be less pronounced than those observed in Figure 2. The resonance frequency is the most important parameter in a resonator as stated by Chen et al. in Chapter 2.3 “Microwave Resonance” [39]. Notwithstanding, the amplitude, bandwidth, phase and Q-factor are still used in the statistical analysis of the measurements.

### 2.2. The Split-Ring Resonator

**Figure 3:**
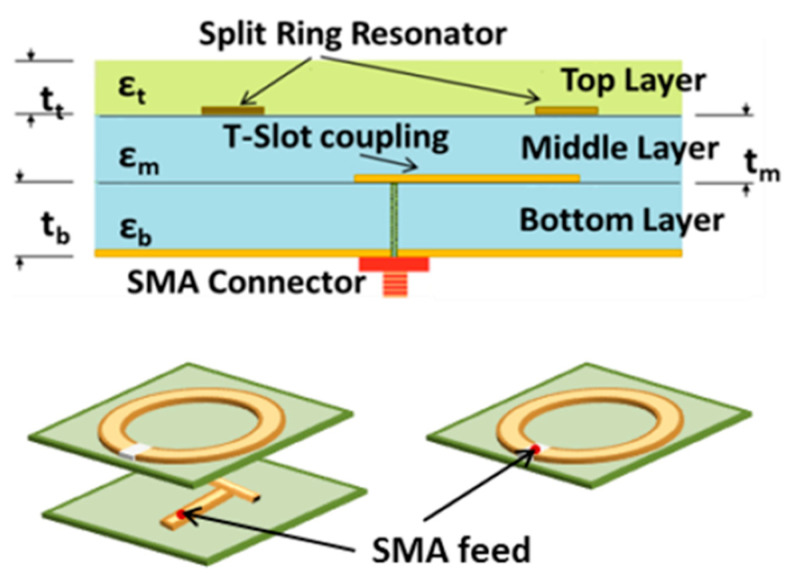
Illustration of the internal structure of the SRR [31].

**Figure 4:**
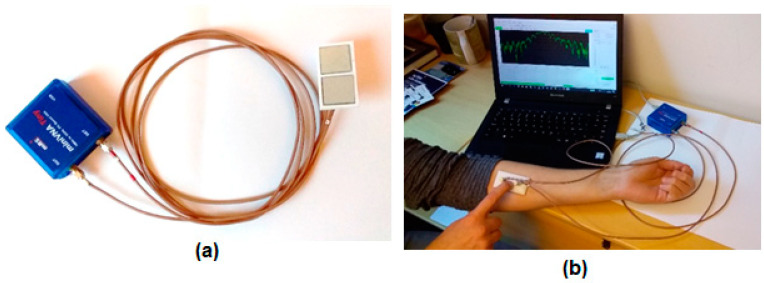
Photographs of the miniVNA and the split-ring resonator (**a**) and measuring the muscle mass on the ventral side of the lower arm (**b**).

The first sensor we developed consisted of two split-ring resonator microwave sensors connected to a portable network analyzer. Similar SRR sensors have been studied in various applications, for the feasibility of using them as biosensors [40,41], as a way to detect biomarkers for stress symptoms [42] and for blood glucose monitoring [21] among others. The network analyzer used in these measurements is a miniVNA Tiny (mini Radio Solutions, 2014, WiMo Antennen 85 und Elektronik GmbH, Herxheim, Germany), in transmission mode. Connecting the network analyzer to a laptop creates a portable system that can be used for measurements. The sensor itself is made up of three layers, the bottom one consists of a Rogers TMM4 substrate, ground plane and a T-shaped feed line. The middle layer is Rogers 6010 substrate with the split-ring resonator itself and the top layer is a superstrate layer of Rogers TMM6. Figure 3 shows an illustrative schematic of the different layers of the SRR. Previous works have used this sensor [31,43,44] to analyze bone healing in patients with lower extremity trauma and patients undergoing surgery in treatment of craniosynostosis. A similar system was presented in those works using a similar hardware configuration but operating with only one sensor in reflection mode. For the new implementation, we captured the transmission coefficient. The sensor is designed to have a resonance frequency of about 2.5 GHz so its relevant operating frequency is 2–3 GHz. A photo of the system is shown in Figure 4a with the miniVNA device to the left and the two SRR sensors to the right. Figure 4b shows the system during a test measurement.

The concept of using the SRR to assess muscle quality is validated through simulations and comparison to volunteer measurements. The simulations studied the amount of the E-field which penetrated through the skin and the fat into the muscle layer. Another simulation study tested the sensitivity of the SRR to changes in dielectric properties of the muscle by varying the relative permittivity of it. The simulations were done with both Ansys HFSS and CST having similar open boundary conditions, except for the port layer, and the same accuracy of convergence (maximum delta of 2%), and involves the sensor positioned on a three-layer tissue model including skin, fat and muscle layers stacked on top of each other. The tissue properties are the dispersive data from the IFAC database [45]. Finally, the simulations are compared to volunteer measurements.

### 2.3. New Proposed Microwave Sensors

During the analysis of the clinical data from the SRR measurements, we saw a difference between the volunteers and the group of patients with an SMI value which put them in the risk of having sarcopenia. But, between patients in the risk of having sarcopenia and those not, based on SMI value no significant difference was detected. This could potentially change with more data, but concerns were also raised over the quality of the measurements as they were quite noisy. In the previous works with the SRR, it was used in reflection mode only. Therefore, the need for a sensor that penetrates the muscle tissue deeper became clear. Several different ideas of new sensors were developed. Three new sensors were proposed. Two are improvements over the SRR and one is a newly designed planar bandstop filter.

#### 2.3.1. SRR with Substrate Integrated Waveguides

**Figure 5:**
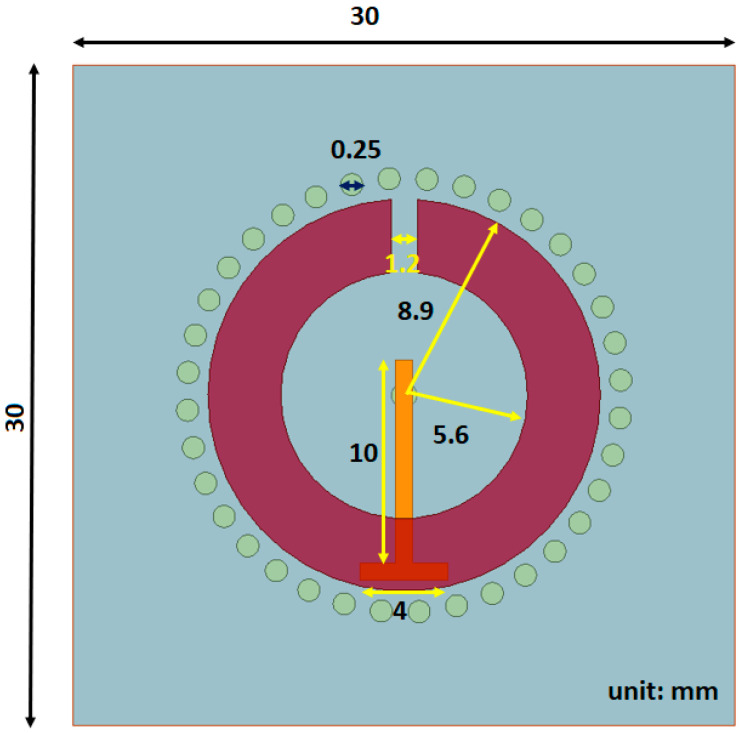
Structure of SRR with SIWs.

The first proposed sensor is a variation to the SRR where small cylinders, with a diameter of 0.25 mm, are inserted into to the substrate layer to act as waveguides, hence the name substrate integrated waveguides (SIW), directing more of the signal into the tissue layer. In other works with SIWs, they are generally in two parallel rows connecting two metal plates [46,47] but in our approach, the SIWs are inserted in a circular configuration around the split ring microstrip. Figure 5 shows the top view, where the circles around the SRR strip are the substrate integrated waveguides, whereas the rest of the sensor is the same as the SRR used in the clinical trials, including the substrate and superstrate materials. Since the overall structure is the same as the SRR, they have the same operating frequency.

#### 2.3.2. Substrate Integrated Waveguides Sensor with Dielectric Resonator (SIW DR)

**Figure 6:**
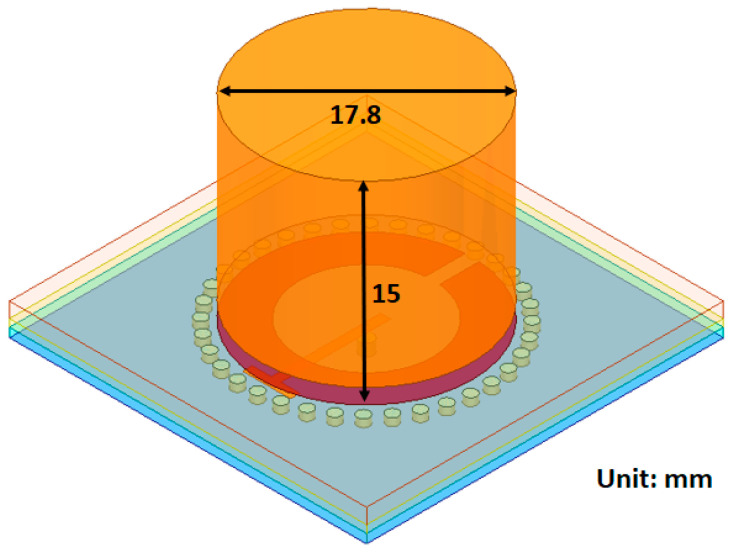
SIW sensor with a dielectric resonator block.

The Substrate Integrated Waveguides, Dielectric Resonator sensor (SIW DR) is another hybrid approach combining the SRR with SIWs together with a block of dielectric resonator on top of the superstrate. The material of the resonator block is a dielectric with a relative permittivity of 38, this is to ensure good matching with the skin layer ($\varepsilon_{r,skin}=38\;@2.45\;\mathrm{GHz}$ [45]). The dimensions of the block are shown in Figure 6. The resonator block is a cylinder covering the split-ring structure but not the waveguides in the substrate. It has a height of 15 mm, which was the optimal height based on a brief simulation study. The SIW DR has the same operating frequency as the SRR and SIW.

#### 2.3.3. Bandstop Filter

**Figure 7:**
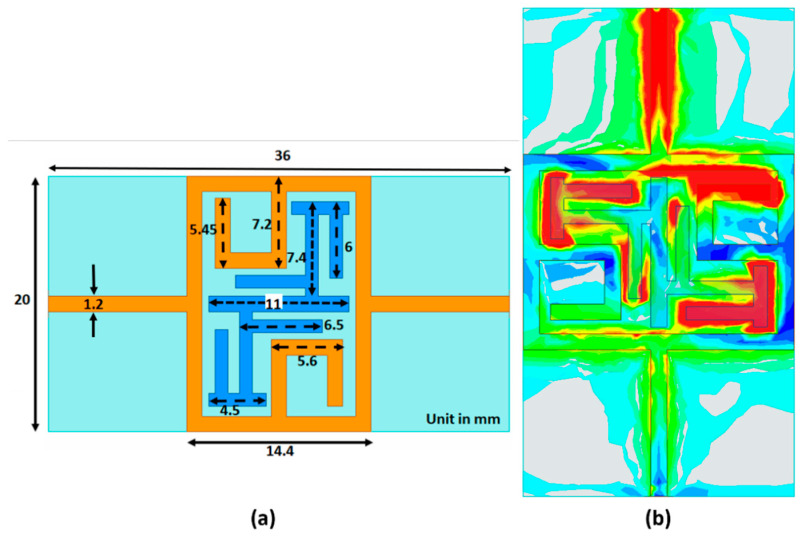
Overview of bandstop sensor structure with dimension (**a**). E-field distribution on the bandstop filter (**b**).

A bandstop microstrip filter is proposed as an alternative design (schematic diagram is shown in Figure 7a). The filter configuration consists of a closed square loop, including a C structure with a modified interdigital π structure which enhances the electric field concentration in the middle of the sensor at the resonance frequency shown (Figure 7b). The color scale indicates the intensity of the E-field which extends from red, as the highest intensity, to green, dark blue, light blue and gray as the lowest intensity. The high electric field concentration is in the middle of the sensor, particularly in the π structures, and emphasizes the field interaction between the human body and the sensor. This subsequently helps to improve the sensitivity and resolution of the sensor [48]. For Figure 7b, the signal propagates from top to bottom. Therefore, the top C structure demonstrates a higher intensity than that for the lower one. The proposed filter is fabricated on low-cost FR4 glass epoxy material with 4.4 dielectric constant, tan δ = 0.02 and thickness of 1.6 mm. Superstrate TMM6 with dielectric constant and loss tangent of 6 and 0.0023, respectively, has been positioned on the sensor’s top layer to enhance the coupling and prevent direct contact to lossy human skin. The operating measurement frequency span for the bandstop filter is from 1.5 to 2.5 GHz.

**Figure 8:**
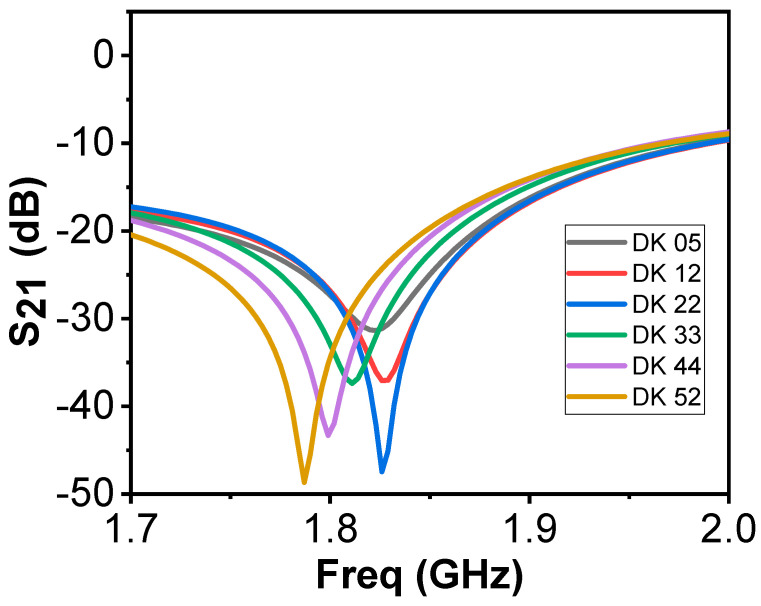
Simulations on one-layer model using the bandstop sensor.

Figure 8 shows the frequency response of the bandstop sensor when simulated on a single layer material. The DK is varied from 5 to 52, at a total of 6 different points, with different interval dielectric step widths in between. We observe significant shifts in the resonance frequency, but also variations in bandwidths as a function of the DK (Q-factor). The Q-factor of the new resonator will also be used in the data analysis. Comparing this to the simulations of the previous design (Figure 2), the bandwidths were similar for all cases, rendering the Q-factor irrelevant for analysis.

**Figure 9:**
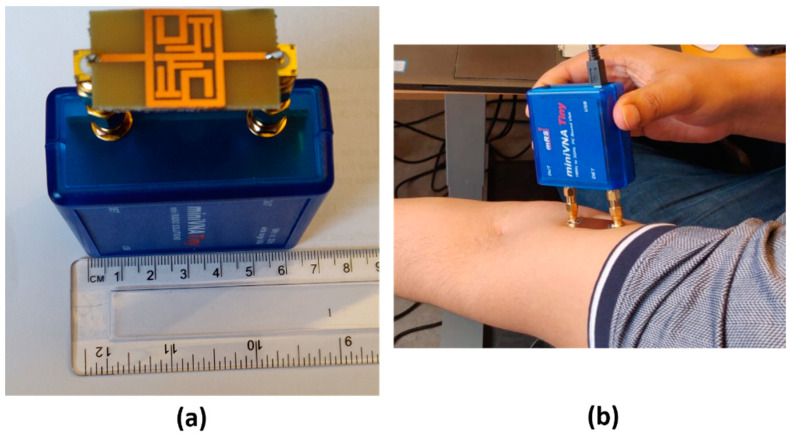
The bandstop sensor without superstrate layer (**a**). The bandstop sensor used in a test measurement (**b**).

In Figure 9a, the produced bandstop sensor is shown connected to the miniVNA Tiny without the superstrate layer, to show the printed bandstop pattern. Figure 9b shows the bandstop sensor in a test measurement.

### 2.4. Simulation Studies of New Microwave Sensors

The new sensors were evaluated via simulations using Computed Simulation Technology (CST) [49], a software package for electromagnetic simulations. This allowed us to study how their frequency responses change with respect to variations in the fat thickness and permittivity of muscle tissue. While examining the thickness of adipose tissue in different parts of the body, the fat thickness in the anterior thigh 8 mm ± 6 [50]. We tested the signal penetration characteristics into the muscle layer at a 1mm depth. This is performed for 2, 8, 14, 30 and 40 mm subcutaneous fat thickness. The three smallest thicknesses cover one standard deviation of the average thickness in the thigh (8 ± 6) [50]. The two larger thicknesses (30 and 40 mm) are used to validate behavior in more extreme cases. To test the sensitivity of the sensors, two different studies were performed. One where the fat thickness is kept constant at 8 mm and the muscle permittivity is varied from 12 to 52 (step width: 10) in order to examine the effects due to changes in the muscle properties. The second is to study the effect of the fat thickness, which is varied from 2 to 40 mm (step width: 1 mm). For each step, the difference in resonance frequency between normal muscle tissue (DK 52) and deteriorated muscle is calculated (DK 12).

The adaptive mesh refinement routine was used to determine a level where an accuracy with a maximum delta of 2% was achieved. The mesh density varied depending on the sensor used, but for all of them it was between 1 and 2 million mesh cells for the smallest model of 2 mm fat. The number of cells subsequently increases for larger fat thicknesses. Boundary settings for the software were open and in all directions except above the sensor which is open (add space), which means a vacuum layer is added between the model and boundary. This implies that the tissues are infinitely extended in the lateral direction and the muscle is also infinitely extended in the medial direction. The tissue model is a simple box model with three layers, skin, fat and muscle. The skin is kept constant at a 2 mm thickness, while the fat thickness ranges from 2 to 40 mm, depending on the study. The muscle is set to 10 mm, albeit the boundary condition imply that the muscle is infinitely extended. The dielectric properties for skin and fat are the dispersive data from the IFAC database [45]. For the muscle, the relative permittivity is given by a constant that varies depending on the simulation—i.e., whether it is normal or deteriorated tissue.

### 2.5. The Clinical Pilot Study with the SRR

A clinical pilot study at the Maastricht University Medical Center (MUMC+) was performed to evaluate the proposed technique of the split ring resonator. Adult patients who underwent an abdominal CT-scan during their visit to the emergency department were eligible for inclusion. Screening took place between February and March 2020. Patients were included when above the age of sixteen, mentally competent, participation had no interference with further needed treatment or diagnosis, and when CT scan and MAS measurements could both be performed within 24 h. Patients who were mentally incompetent, missing one or both legs, or who were unable to grab the handgrip meter were excluded from this study.

The muscle surface area was assessed by abdominal CT on the third lumbar spine vertebra (L3) and the skeletal muscle index (SMI) was calculated using those scans in a standardized manner [51]. In addition, BIA measurements were performed the same day. MAS measurements were obtained in the mid-thigh region (both legs, three repetitions). The mid-thigh area corresponds well to whole-body muscle mass [52] and generally, the fat layer in the mid-thigh region is thin compared to other areas of the body, with a large muscle underneath [53]. In addition to MAS and BIA, circumference measurements, hand grip strength (HGS) and SARC-F questionnaires were obtained for each patient.

The measurements were non-invasive, had no adverse effects and took a short amount of time. Therefore, the patient burden was negligible. We received permission from the Medical Ethics Review Committee from the Maastricht University Medical Centre Ethics Board for this study.

Apart from the patients, measurements from healthy volunteers were also performed and included in this study. These volunteers were measured using the MAS device. Leg circumference was measured, SARC-F questionnaire was filled in and information including age, height and weight were collected.

The sensors of the MAS device were placed on the upper leg and connected to the miniVNA and to the computer (Asus, Windows 10). The researcher placed an elastic band on the upper leg, 10 cm above the patella on the thigh. Four separate measurements were performed with the sensors at different spacing between them. The first measurement was carried out with the electrodes right next to each other on the lateral side of the upper leg. For the second measurement, the electrodes were placed 2 cm apart from each other in the elastic band. The third measurement was carried out with the electrodes 5 cm apart from each other in the elastic band. The last measurement was executed with the electrodes 5 cm apart from each other parallel on the lateral side of the upper leg. The same strategy was repeated on the contralateral side.

Since there was concern regarding quality in both measurements with the sensors placed 5 cm apart (data not shown), these measurements were discontinued after 25 exams. Measurements with the split ring resonators’ opening in line with each other gave better results than at a 90-degree angle. Therefore, the electrodes were marked, and subsequent measurements were conducted with the split ring resonators opening in line with each other.

A number of parameters were derived from each dataset from the MAS device measurements, as listed in Table 1. These measurements were used in the statistical analysis.

To validate the frequency measures with the gold standard SMI, we calculated the SMI values of the patients. These threshold values for muscle quality based on their SMI are 52.4 cm^2^/m^2^ for males and 38.5 cm^2^/m^2^ for females [51]. The patient data was divided into three different groups based on these threshold values: Group 1 for SMI < 0.8*Threshold, Group 2 for 0.8*Threshold < SMI < Threshold and Group 3 for SMI > Threshold. The healthy volunteer data were referred to as Group 4. They are assumed to be healthy and have a SMI above the threshold value for good muscle quality for their respective gender. The BIA analysis generates several parameters that are given as an absolute value or as a percentage of the bodyweight. In those cases, the percentage value was used in the analysis. There are 43 parameters in total when combining the MAS and BIA parameters. All parameters identified in both MAS and BIA measurements were weighted equally in the analysis and each parameter is adjusted using a standard z-score normalization with mean 0 and standard deviation of 1 to align the parameters on a similar scale making it easier to compare. The best parameters for the MAS data were identified by testing all available combinations of parameters, and sorting after their resulting *p*-value.

To evaluate the distribution of different groups, we ran the ANOVA test. The goal was to identify statistically significant differences between the groups. When the *p*-value was below the significance level of 0.05, the groups were categorized as different. The Tukey–Kramer test was run on the results that determined which groups were statistically significantly different from other groups [54,55].

### 2.6. Healthy Volunteer Study Using the Bandstop Filter and SRR

The bandstop filter and the SRR were used in a study involving healthy volunteers. The purpose of this was to validate that the developed bandstop filter gave the expected measurement results based on the simulations and to compare the performance of the bandstop to the SRR. The measurements were taken 10 cm above the patella in the medial direction, the same position in which the measurements in the previous clinical study were performed. The volunteers also underwent ultrasound measurements measuring the skin, fat and the rectus femoris muscle thickness. BMI and leg circumference were also measured, and age information was collected.

## 3. Results

### 3.1. “Proof-of-Concept” Simulations

**Figure 10:**
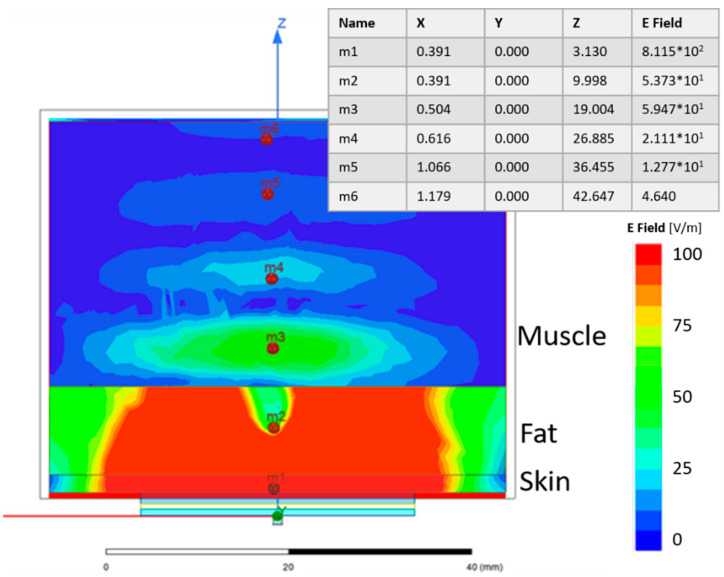
Electric field distribution of the SRR on a 3-layer model. The table shows the strength of the numeric values of the electric field at six selected points. For the included table X, Y and Z are given in mm and E field in V/m.

A simulation study was performed to evaluate the signal strength penetrating through the skin and fat layers and into the muscle layer. Figure 10 shows the electric field distribution of a single SRR on a 3-layer model, where the first layer is 2 mm skin, the second layer is 10 mm fat and the third is 30 mm muscle. Even though a large amount of the electric field is confined to the skin and fat layers, the E-field in the muscle is still reasonably strong. This is shown at point m3 in Figure 10.

Lower quality muscle is expected to have more fatty tissue than healthy muscle tissue [3]. Given that the relative permittivity of fat is lower than that of muscle (5.28 and 52.73 at 2.45 GHz, respectively [45]), the lower quality muscle will have a lower relative permittivity compared to healthy muscle tissue. Simulations were performed to test the frequency response changes, where the relative permittivity of the muscle is subtracted by a constant.

**Figure 11:**
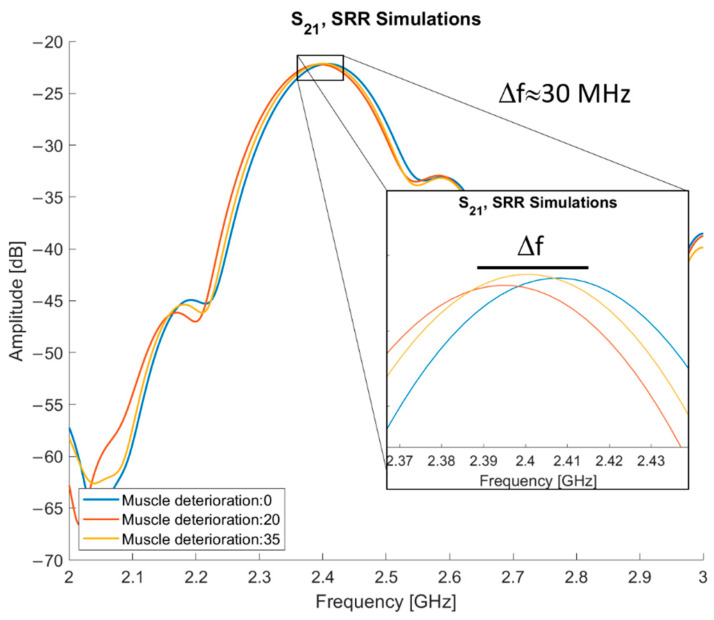
S_21_ of simulations with normal and deteriorated muscle tissue.

The results of the simulations using varying muscle quality are presented in Figure 11. By subtracting 20 and 35 from normal muscle, three different levels of muscle quality are tested: normal muscle and two levels of deterioration. The entry “muscle deterioration: 0” is normal muscle. The maximum difference in resonance frequency (f_res_) is between normal muscle and muscle deterioration 20, they have an approximate 30 MHz difference, with normal muscle tissue having the higher f_res_. The f_res_ for deterioration of 35 is placed in between the other two, approximately 15 MHz below the f_res_ of normal muscle.

**Figure 12:**
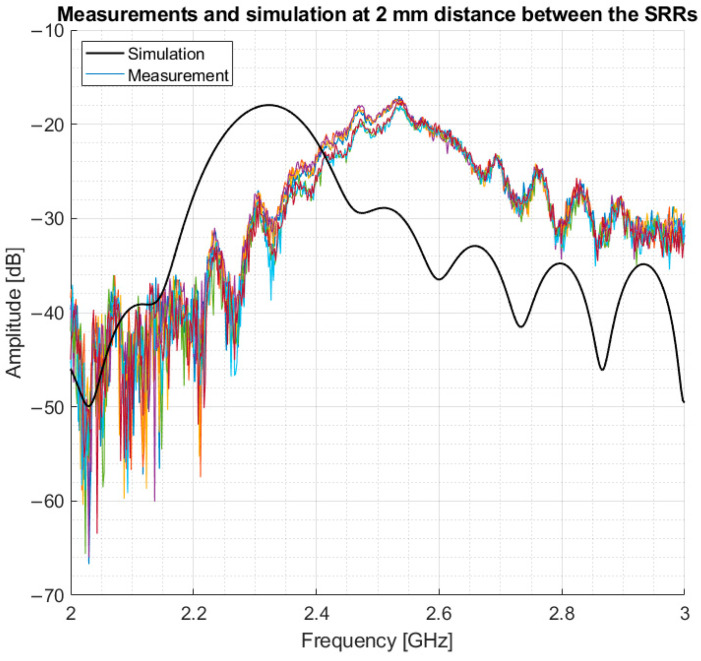
Comparison between volunteer measurements and simulation.

Following the simulations, measurements were performed in a volunteer, and the resulting data were compared to simulations on a 3-layer model. The measurements were performed in the mid-thigh, the same area modelled in the simulations, to evaluate how comparable the simulations are to measurements. This model consisted of 2 mm skin, 5 mm fat and infinitely extended muscle. The results showed that the simulation had a lower resonance frequency in comparison to the volunteer. However, the same amplitude and a similar bandwidth of 5 dB around resonance were found. This is shown in Figure 12.

The results from the simulations, as depicted in Figure 10 and Figure 11, validate the sensor as being able to detect difference in muscle quality with sufficient penetration depth. In addition, Figure 12 shows that the volunteer measurements and simulations are comparable as well; this justifies moving to clinical trials.

### 3.2. Statistical Analysis of Clinical Study Data

20 patients, 11 females and 9 males with an age of 61.7 ± 13.6 (range 37–81 years), and 25 healthy volunteers, 9 females and 16 males with an age of 27.7 ± 13.4 (range: 18–66 years), have been recruited. During the study, we realized that the split in the internal ring needed to be oriented in the same direction to measure the transmitted signal. This behavior was confirmed via simulations and test measurements; it happens because the split in the microstrip ring creates a non-uniform radiation pattern so if the orientation is incorrect, the signal received will be much lower. For our case, it means if the split in the rings is not aligned, the transmitted signal was below the noise floor and those measurements could not be used in the analysis. In total, data from only 9 patients and 21 volunteers are included in the study. Moreover, BIA data for volunteers were not present. After the data reduction due to the mentioned restrictions, there is only one measurement in group 1. Groups 2 and 3 each have five measurements and group 4 with volunteers has 26 measurements. Due to the small sample size, group 1 was excluded from statistical analysis.

The ANOVA assumptions of the samples being normally distributed, and the homogeneity of variance were all met, for both the MAS and BIA data, except for one parameter. The slope parameter from the MAS data failed the normal distribution test. However, the *p*-value of this parameter remains fairly high, 0.0036. Because the rest of the parameters pass the test, this is not an issue. To test if the samples were normally distributed, the Kolmogorov–Smirnov test was used. For the homogeneity of variance, the Brown–Forsythe test was used. The sample cases are all independent of each other as they are all separate measurements.

**Figure 13:**
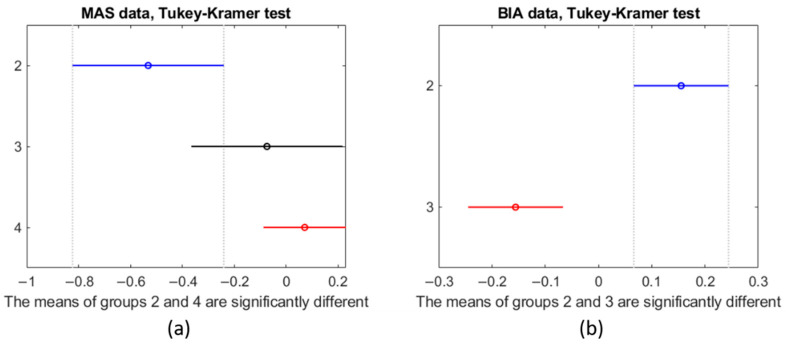
Results of the Tukey–Kramer test of the (**a**) MAS data and on the (**b**) BIA data. The y-axis indicates the group number.

The data from the MAS device showed different values for all groups (F-test, *p*-value: 7.8 × 10^−3^) when considering the six parameters. When considering all six parameters derived from the MAS data at the same time, group 2 and group 4 (F-test, *p*-value: 2.5 × 10^−3^) were significantly different. Group 3 did not differ from Group 4 (F-test, *p*-value: 0.44) and neither did group 2 and group 3 (F-test, *p*-value: 0.088). The Tukey–Kramer test validated the results of the F-Statistic test. Figure 13 shows the mean and standard deviation for each group as vertical lines. The value of the x-axis is that of the z-score normalization. In this visual representation, lines that do not overlap are significantly different from one another. As can be seen in Figure 13a, there is a clear trend that shows that, when considering all six parameters, a lower SMI implies a more negative z-score.

Each possible subset of the six parameters was evaluated resulting in a total of 64 combinations. A subset of the parameter’s resonance frequency, unwrapped phase from 1 GHz and 2 GHz gave the best F-Statistic score (F-test, *p*-value: 3.13 × 10^−5^), which was found in all groups. The second-best parameter set was the same as the best but included the bandwidth of 5 dB (F-test, *p*-value: 1.46 × 10^−5^). None of the best subsets, in terms of F-score, included the slope of the phase with the rest of the parameters giving the fifth best score (F-test, *p*-value: 4.31 × 10^−5^).

**Figure 14:**
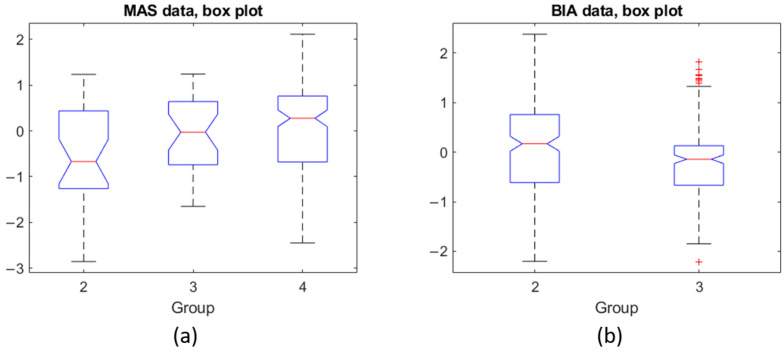
(**a**) Box plot for each group of the MAS data, (**b**) Box plot for the two groups of BIA data.

Figure 14 shows the box plots for MAS (subplot a) and BIA (subplot b). The y-axis corresponds to the z-score normalization. For the MAS data, all groups show a similar range from 25th percentile (Q1) to 75th percentile (Q3). Group 4, however, shows that the extreme values are further from the mean than the other groups. The BIA data for group 3 shows that Q1 and Q3 are close together indicating a tight grouping of the values in that group but with a number of outliers. Group 2 is similar to the boxes for the MAS data.

Regarding BIA, while we could not compare to group 4 because the measurements using the BIA were not performed on the volunteers, group 2 and group 3 are significantly different (F-test, *p*-value: 0.0007).

### 3.3. Results of the New Sensors

#### 3.3.1. Electric Field Strength

**Figure 15:**
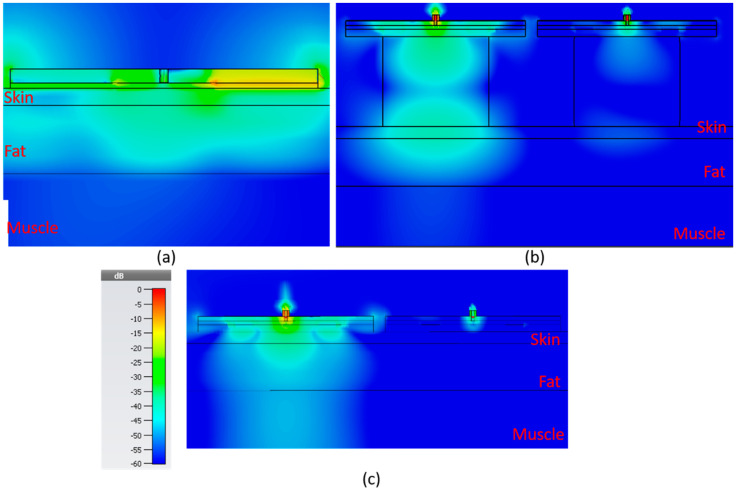
E-field for the 3 new sensors, 2 mm skin, 8 mm fat, 10 mm muscle (**a**) bandstop at 1.85 GHz, (**b**) SIW DR at 2.45 GHz and (**c**) SIW at 2.45 GHz. Color scale applies for all subfigures.

In Figure 15, the electric field distribution is shown for the sensors, in the order of bandstop sensor, SIW with resonator block and SIW without resonator block. The color scale ranges from −60 to 0 dB. For the SIW sensors, both with and without resonator block, there are two sensors side by side 2 mm apart.

In Table 2, the strength of the electric field for each sensor is shown 1 mm into the muscle layer directly underneath the sensor for five different fat thicknesses: 2, 8, 14, 30 and 40 mm. The value is the root mean square (RMS) value over the full period. The table suggests that the SIW sensor without a resonator block would be the best of the alternatives as it displays the highest amplitude in the electric field for all of the different fat thicknesses.

#### 3.3.2. Resonance Frequency and Q-Factor Analysis

To analyze and validate the sensors, they were simulated using CST while varying fat thickness and dielectric constant of the muscle tissue. Table 3 shows the resonance frequency from simulations performed with 2 mm skin and 8 mm fat. Five different values for the DK of the muscle are used, 52 for normal muscle and subsequently subtracting by 10 for each variation to create four levels of deteriorated muscle, 42, 32, 22 and 12, respectively. Each sensor has two columns f_res_ shows the resonance frequency in GHz and Δf_res_ is the difference compared to normal muscle (DK 52). The simulations are generated from 1–3 GHz in steps of 0.5 MHz. The results in the table show that the SIW DR and the bandstop are the two sensors with the highest measurement difference with respect to the full muscle property range which implies that they are the most sensitive to changes in the muscle dielectric constant. Table 4 shows the Q-factor calculated at the associated 3 dB bandwidth for the same data in which the results in Table 3 were produced. The table shows that there are only minor differences as the muscle properties change except for the bandstop sensor which shows quite large variations, which is to be expected considering the graphs in Figure 8.

**Figure 16:**
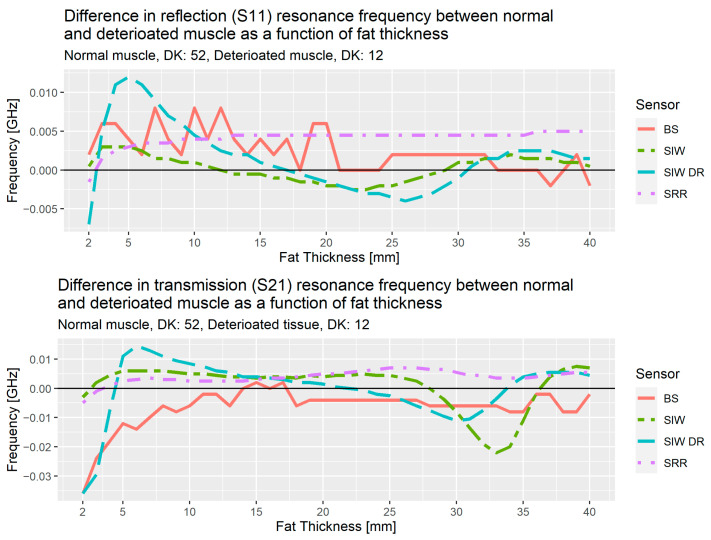
Difference in resonance frequency for reflection and transmission as a function of fat thickness.

Figure 16 shows the results of the analysis from the simulations for the three sensors, bandstop filter (BS), SIW and SIW DR, and the results from the SRR, the sensor used in the initial studies. These results are the difference in resonance frequency for normal muscle tissue (DK: 52) versus deteriorated muscle tissue (DK: 12), the upper plot is for reflection, S_11_, and the lower is for transmission, S_21_. As an example, SIW DR at 5 mm for reflection has the value 0.012 GHz, this means the resonance frequency for normal muscle tissue is 0.012 GHz higher than the resonance frequency for deteriorated muscle tissue.

Up to 10 mm of fat thickness, the transmission coefficient for the bandstop shows a near linear decrease with the largest difference at 2 mm and decreasing until 10 mm. This shows that the bandstop filter is a good option for people with subcutaneous fat layer less than 10 mm.

In the region spanning from 5–30 mm for the SIW DR, we observe a linear behavior as the difference between normal and deteriorated muscle decrease from about 0.01 GHz at 5 mm for both reflection and transmission, to −0.004 GHz for reflection at 25 mm and to −0.01 GHz at 30 mm for transmission.

The results from these simulations allow us to propose a solution consisting of two different sensors. If the fat layer has a thickness of less than 10 mm, we use the bandstop sensor, otherwise the SIW DR will be used. We also note that for fat thicknesses above 30 mm, SIW shows an interesting rapid decrease and then increase that needs further investigation.

### 3.4. Pilot Study of Healthy Volunteers Comparing the SRR and the Bandstop Filter

**Figure 17:**
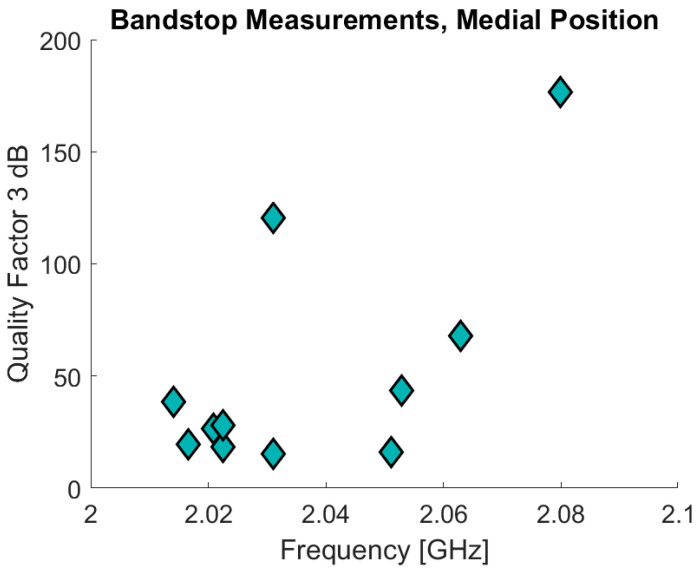
Bandstop and SRR volunteer measurements.

The bandstop filter has been tested in a pilot study that also involved ultrasound measurements on the fat and skin thicknesses. Eleven of the volunteers measured were included in the pilot study of which 10 also underwent the ultrasound measurements. The volunteers were also measured using the SRRs. The age of the volunteers was 30 years ± 7.8 (range: 23–50) and BMI values are 22.3 ± 1.9 (range: 19.88–24.98). The data are analyzed in a similar manner compared to the data from the previous clinical study. In Figure 17 the initial results are presented with the resonance frequency on the x-axis and the Q factor on the y-axis for the bandstop measurements in the medial position. The measurements are for the sensor positioned 10 cm above the patella in the medial direction, the same position as used in the clinical trials. The Δf_res_ for the bandstop is 66 MHz (min: 2.014 GHz, max: 2.08 GHz) and for the SRR measurements Δf_res_ is 830 MHz (min: 2.17 GHz, max: 3.00 GHz). The SRR data are excluded from Figure 17 because most of the associated measurements consist primarily of noise. This noise is the reason why the maximum resonance frequency for one of the measurements is the maximum measurement frequency, 3 GHz. This also contributes to why the SRR measurements demonstrate such a high Δf_res_. The bandstop data also present a high Q-factor variance: ranging from a minimum of 15.27 to a maximum of 176.49.

**Figure 18:**
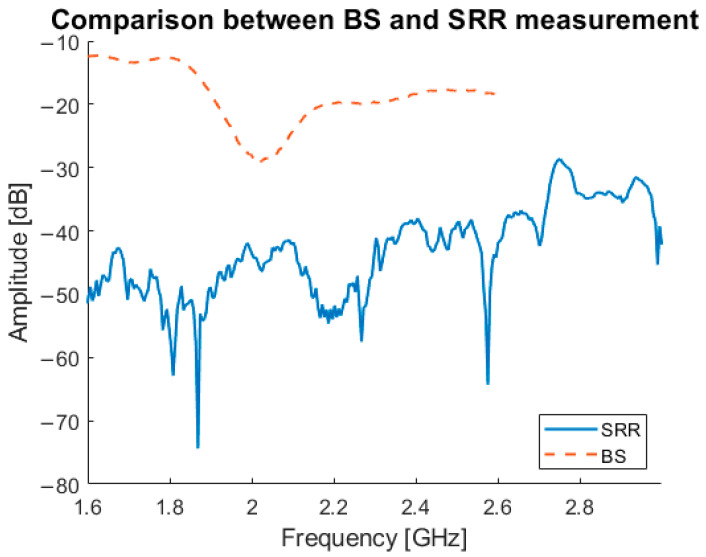
Comparison between the SRR and bandstop sensor.

In Figure 18, a comparison between the newly developed bandstop sensor, and the SRRs is shown. The graph illustrates the difference between transmitted signal from the bandstop sensor and the SRR, with the bandstop sensor showing a clear signal without interference. The SRR measurement shown is one of only two measurements that showed a good result; the signals for of the remainder were always below −40 dB and effectively below the noise floor.

## 4. Discussion

The use of microwave-based sensing as a tissue analysis system is recently gaining interest due to advantages such as non-ionizing radiation and non-invasiveness. Since sarcopenia is a progressive skeletal muscle disorder that affects the quality of life, increases risk of falls and fractures and increases mortality, the need for a muscle quantification tool that is widely available, user-friendly, efficient, non-invasive and inexpensive is high [3,4,5].

This study shows the development of a microwave sensor for quantification of muscle mass and muscle decline in diseases like sarcopenia. Our simulation studies have shown substantial improvements in the new version of the sensor; however, clinical validation is still ongoing. Still, to the best of our knowledge, the idea to assess muscle quality via microwave characterization is the first of its kind.

Although the project is still in its initial stage and more data are yet to be analyzed, this article provides further insight into microwave sensing. In the first phase, the SRR sensor was clinically tested, showing a significant correlation with CT-derived data, but a lower sensitivity compared to BIA data. Additional evaluations indicated that the SRR is not suitable for muscle mass quantification due to limited penetration depth. Although the SRR had the potential to distinguish between patients it can be improved with a sensor optimized for penetrating deeper and thereby acquiring increased information from the muscle layer. Therefore, new alternative sensors were produced for comparison with the SRR. This is a proof-of-concept study, and it is essential to note that we assess muscle quality and do not diagnose sarcopenia.

The SIW DR was developed as one of the alternatives for the SRR. For the SIW DR, it is important to have both the reflection and transmission coefficient data. However, the currently used miniVNA together with vna/J software are unable to capture both measurements simultaneously. To use the SIW DR efficiently, this will need to be resolved; either by using a different VNA or custom software that can capture both coefficients simultaneously. Therefore, at this point, the preferred sensor is the bandstop sensor, although additional measurements in a wider range of tissue composition need to be obtained.

For the simulation studies in the second phase, a DK of 12 was used for deteriorated muscle. Assigning the deteriorated muscle a DK of 12 implies a relatively large fraction of deteriorated muscle consists of fat, which has a DK of 5. This is relatively low, since normal tissue has DK 52. However, the point of this simulation study is a proof of concept for comparing the different alternatives, which has been done in this study. In future studies, different DK values will be added to determine the sensitivity of the MAS sensor in detecting small variances in muscle quality, but it is currently unknown what threshold in terms of DK would be considered low muscle quality. Table 2 demonstrates the premise that the substrate integrated waveguides help create a signal more directed into the tissue as it has the highest level of E-field penetrating into the muscle. The results from Table 4 and Figure 17 shows that the bandstop sensor has a much higher Q-factor than the other sensors, which means it should be more sensitive to changes in the muscle properties.

We take into account several important considerations when designing sensors exploiting frequency variations as the primary metric as is the case with those used here. The compact sensors must be designed such that their fundamental resonance is in the frequency region of interest [33]. The external environment needs to be accounted for in that it can unintentionally affect the permittivity—i.e., temperature and humidity. However, in our case, such factors are essentially negligible since we position the sensors directly on skin and let the system thermally stabilize so subsequent variations in temperature and humidity are minor. To mitigate the effects of cross-sensitivities sensors based on symmetry truncation and differential-modes can be employed [56].

The initial version of the sensor, the SRR, did show differences between patients and volunteers with different SMI ranges, but those differences are not yet large enough to claim that MAS identifies low muscle quality in its current iteration. Due to this limitation, the need for an alternative sensor more attuned to deeper sensing in the muscle layer arose. The SIW and bandstop sensors were then evaluated via numerical simulations with the purpose of testing how well the signal from those sensors penetrates into the muscle layer and if they are suitable to estimate muscle quality or not. The comparison between the bandstop and SRRs shows that the bandstop produces reliable measurements whereas the SRR measurements are very noisy. This noise in the SRR data is why the Δf_res_ is so much larger compared with the bandstop data. This gives the bandstop sensor a clear advantage over the SRR sensor. From the simulation results a solution using two different sensors dependent on the fat thickness is proposed: the bandstop sensor if the fat thickness is below 10 mm and the SIW DR if it is above 10 mm. This limitation has to be taken into account when translating these data to a clinical setting, since at this point the correct sensor selection depends on the fat thickness as measured by ultrasound. The results in Table 3 confirm that the SIW DR and the bandstop sensors are the most sensitive where they demonstrate the largest differences between normal and deteriorated muscle.

The volunteer measurements using the bandstop sensor confirm that the sensor is working as intended, especially regarding the agreement in high Q-factor and resonance frequency between the measurements and simulations. The volunteers had a narrow range in body composition, indicated by their age, BMI and ultrasound measurements. Accordingly, the MAS measurements presented a correspondingly narrow range, supporting the hypothesis that when testing over a wider range of body composition, MAS will be able to identify this correctly. One limiting factor to this study is that the gold standard, SMI, was not measured. That data could have potentially assessed how the bandstop frequency response correlates to different SMI values.

## 5. Conclusions

This article describes the development of a microwave-based sensing technique for quantification of muscle mass and muscle quality. Following clinical tests with a prototype version of the sensor, the penetration depth of the microwave signal was identified as the limitation in implementation. Therefore, three new sensors have been developed: two with integrated waveguides and one with a bandstop filter. Simulation tests, with the SIW DR and the bandstop filter, and initial human measurements, with only the bandstop filter, show promising results compared to the previous version, using the SRRs, but clinical validation is still needed.

Further work is needed to fabricate a MAS device that can be used in clinics. The SIW DR need to be constructed and tested on artificial tissue emulating phantoms, volunteers and in the clinical setting. Further validation of the bandstop is also needed. These need to be performed so a similar statistical analysis, as in the first clinical study, can be processed to evaluate the characteristics on patients. Testing the performance of the new sensors on phantoms creates a setting where more control can be had over the fat thickness and muscle properties. The ultimate goal is to develop an algorithm that can provide an estimate of the muscle quality from the sensor measurements. This can then be used in a purpose-built application that allows a Raspberry Pi computer to perform and transmit a measurement to a remote server. This is currently in development which will allow the MAS to become a wireless handheld device.

## Figures and Tables

**Table 1:** Parameters from the MAS measurements.

Parameter	Description
Resonance frequency	The frequency at which the amplitude is at its maximum
Amplitude	The amplitude at the resonance frequency
Bandwidth of 5 dB around resonance	The frequency ranges in which the amplitude is above amplitude at resonance—5 dB
1 GHz	Unwrapped phase from 1 GHz at resonance
2 GHz	Unwrapped phase from 2 GHz at resonance
Slope	Slope of the phase around resonance.

**Table 2:** Strength of electric field, in dB (μV/m), 1 mm into muscle layer.

Fat Thickness in mm\Sensor	SRR	SIW	SIW DR	Bandstop
2	−41.2	−35.7	−44.3	−49.8
8	−53.0	−47.3	−54.3	−54.1
14	−61.6	−47.5	−59.8	−59.4
30	−63.8	−49.8	−61.1	−65.5
40	−68.1	−51.0	−65.2	−67.2

**Table 3:** Resonance frequency and difference in resonance frequency, compared to normal muscle for the sensors, values in given in GHz.

	SRR		SIW		SIW DR		Bandstop	
Muscle DK	f_res_	Δf_res_	f_res_	Δf_res_	f_res_	Δf_res_	f_res_	Δf_res_
52	2.3865		2.38		2.4725		1.828	
42	2.384	0.0025	2.38	0	2.464	0.0085	1.83	−0.002
32	2.3825	0.0040	2.38	0	2.468	0.0045	1.836	−0.008
22	2.3825	0.0040	2.378	0.002	2.46	0.0125	1.832	−0.004
12	2.3835	0.0030	2.376	0.004	2.4615	0.011	1.84	−0.012

**Table 4:** Quality factor at 3 dB for the sensors with varying muscle DK.

Muscle DK\Sensor	SRR	SIW	SIW DR	Bandstop
52	22.51	15.26	11.39	182.80
42	22.49	15.26	6.48	228.75
32	22.27	15.26	6.52	459.00
22	22.27	15.24	6.51	305.33
12	22.28	15.23	6.64	306.67

## Data Availability

The data presented in this study are available on request from the corresponding author. The data are not publicly available due to being data collected in clinical trials from patients.
